# In Vitro Activity of Ibrexafungerp against a Collection of Clinical Isolates of *Aspergillus*, Including Cryptic Species and Cyp51A Mutants, Using EUCAST and CLSI Methodologies

**DOI:** 10.3390/jof7030232

**Published:** 2021-03-20

**Authors:** Olga Rivero-Menendez, Juan Carlos Soto-Debran, Manuel Cuenca-Estrella, Ana Alastruey-Izquierdo

**Affiliations:** 1 National Centre for Microbiology, Mycology Reference Laboratory, Instituto de Salud Carlos III, 28222 Madrid, Spain; olgariveromenendez@gmail.com (O.R.-M.); juanc.soto@isciii.es (J.C.S.-D.); mcuenca-estrella@isciii.es (M.C.-E.); 2 Spanish Network for Research in Infectious Diseases (REIPI RD16/CIII/0004/0003), Instituto de Salud Carlos III, 28222 Madrid, Spain

**Keywords:** antifungals, ibrexafungerp, *Aspergillus*, *cyp*51A, cryptic, EUCAST, CLSI

## Abstract

Ibrexafungerp is a new orally-available 1,3-β-D-glucan synthesis inhibitor in clinical development. Its in vitro activity and that of amphotericin B, voriconazole, and micafungin were evaluated against a collection of 168 clinical isolates of *Aspergillus* spp., including azole–susceptible and azole–resistant (Cyp51A mutants) *Aspergillus fumigatus sensu stricto* (*s.s.*) and cryptic species of *Aspergillus* belonging to six species complexes showing different patterns of antifungal resistance, using EUCAST and CLSI antifungal susceptibility testing reference methods. Ibrexafungerp displayed low geometric means of minimal effective concentrations (MECs) against *A. fumigatus s.s.* strains, both azole susceptible (0.040 mg/L by EUCAST and CLSI versus 1.231 mg/L and 0.660 mg/L for voriconazole, respectively) and azole resistant (0.092 mg/L and 0.056 mg/L, EUCAST and CLSI, while those for voriconazole were 2.144 mg/L and 2.000 mg/L). Ibrexafungerp was active against most of the cryptic species of *Aspergillus* tested, yielding MEC values only comparable to those of micafungin. Nevertheless, this new compound exhibited a moderate activity against *A. ustus* complex species, MECs ≥ 0.5 mg/L against *Aspergillus insuetus* and *Aspergillus keveii* strains, and was inactive against the *Aspergillus alliaceus* isolates tested (MEC_90_s ≥ 16 mg/L). All in all, ibrexafungerp shows encouraging in vitro results against cryptic species of *Aspergillus* and azole–susceptible and azole resistant strains of *A. fumigatu*s, some of which are difficult to treat using the available therapeutic options.

## 1. Introduction

*Aspergillus* species are ubiquitous filamentous fungi that can cause a wide range of infections that are increasing their incidence and threatening the survival of their hosts, especially immunocompromised patients [1]. While *Aspergillus fumigatus* is responsible for most of the fatal cases of invasive fungal disease, the availability of molecular identification tools has led to the description of cryptic species that had previously been misidentified by classical methods [2]. Closely related cryptic species are gathered in species complexes. Their importance in the clinical setting, in which they have been reported to have a prevalence of up to 19% in several studies [3,4,5], and up to 29% in a recent one [6], is determined by the low susceptibility they generally show against antifungals [3]. The fact that this can lead to poor clinical outcomes [7], together with the toxicity and the interaction with other concomitant medications that these drugs can show [8], evidences the necessity of developing antifungals with new mechanisms of action that help to overcome the limitations of existing clinical drugs.

Ibrexafungerp, formerly SCY–078, is the most representative compound within the triterpenes, a new class of antifungals. This semisynthetic derivative of enfumafungin inhibits the fungal β-(1,3)-D-glucan synthase as echinocandins do, although it is structurally different from those [9] and has an overlapping but independent binding site to the enzyme that generates an alternative drug–enzyme interaction [10]. Its in vitro activity has been successfully assessed against *Candida* spp., including echinocandin resistant and multidrug–resistant *Candida glabrata* and *Candida auris* [11,12,13], and several *Aspergillus* species, among which azole or echinocandin resistant *A. fumigatus* isolates stand out, although *Aspergillus flavus*, *Aspergillus niger*, and *Aspergillus terreus*, and a scarce number of *Aspergillus glaucus*, *Aspergillus nidulans*, and *Aspergillus westerdijkiae* strains have also been tested leading to promising results [14,15,16,17]. Ibrexafungerp has been reported to display a moderate activity against *Scedosporium* spp. and *Scopulariopsis* spp., although it has also been proved to be ineffective against *Purpureocillium lilacinum*, *Fusarium* spp. or the Mucorales order [18]. The in vivo activity of this compound has been positively demonstrated when orally or intravenously administered to murine models of invasive candidiasis, invasive aspergillosis, and pneumocystosis [9,14,19,20,21,22,23].

The aim of the present study was to assess the in vitro activity of ibrexafungerp and several antifungal comparators against a collection of *Aspergillus* clinical isolates, including azole susceptible and resistant *A. fumigatus s.s.* and cryptic species, using antifungal susceptibility by CLSI and EUCAST reference methods. This report constitutes the first one in determining the activity of this new compound against cryptic species of this genus.

## 2. Materials and Methods

A total of 168 *Aspergillus* strains belonging to six different species complexes were tested: 79 isolates from the *Aspergillus fumigatus* complex (10 azole–susceptible *A. fumigatus s.s.*, 10 azole–resistant *A. fumigatus s.s.* harbouring different mutations in *cyp*51A gene and in its promoter (4 TR_34_/L98H, 2 TR_46_/Y121F/T289A, 1 G54E, 1 G54R, 1 M220I, and 1 M220T), 20 *Aspergillus lentulus*, 10 *Aspergillus fumigatiaffinis*, 10 *Aspergillus thermomutatus*, 10 *Aspergillus udagawae*, 7 *Aspergillus hiratsukae*, and 2 *Aspergillus felis*), 18 from the *Aspergillus terreus* complex (8 *Aspergillus citrinoterreus*, 4 *Aspergillus carneus*, 3 *Aspergillus aureoterreus*, and 3 *Aspergillus hortai*), 20 from the *Aspergillus ustus* complex (15 *Aspergillus calidoustus*, 3 *Aspergillus insuetus*, and 2 *Aspergillus keveii*), 15 from the *Aspergillus circumdatii* complex (10 *Aspergillus ochraceus* and 5 *Aspergillus sclerotiorum*), 20 *Aspergillus alliaceus* from the *Aspergillus flavus* complex, and 16 *Aspergillus tubingensis* from the *Aspergillus niger* complex. All strains were obtained from clinical samples (respiratory, cutaneous, ocular, optical, biopsies, abscesses, blood cultures, and wounds) and identified to species level by standard microscopic morphology and by sequencing the Internal Transcribed Spacer Region of the rDNA as well as part of the β–tubulin gene, following methods previously reported [24]. The Calmodulin gene was also sequenced for those strains identified as part of the Nigri species complex [25], as it has been reported as the best marker to identify strains belonging to this complex at the species level [26].

Antifungal susceptibility testing was performed following the European Committee on Antimicrobial Susceptibility Testing (EUCAST) reference method 9.3.2 [27] and the Clinical & Laboratory Standards Institute (CLSI) M38 [28]. The antifungals used were ibrexafungerp (range 0.03–16 mg/L; Scynexis, Inc., Jersey City, NJ, USA), amphotericin B (range 0.03–16 mg/L; Sigma–Aldrich Quimica, Madrid, Spain), voriconazole (range 0.015–8 mg/L; Sigma–Aldrich Quimica, Madrid, Spain), and micafungin (range 0.004–2 mg/L; Astellas Pharma Inc, Tokyo, Japan).

*Aspergillus flavus* ATCC 204,304 and *A. fumigatus* ATCC 204,305 were used as quality control strains in all tests performed for both methods. Minimal inhibitory concentrations (MICs) for amphotericin B and voriconazole, and minimal effective concentrations (MECs) for micafungin and ibrexafungerp, were visually read after 24 and 48 h of incubation at 35 °C in a humid atmosphere. Geometric mean (GM), MIC_50_/MEC_50_ (MIC/MEC causing inhibition of 50% of the isolates tested) and MIC_90_/MEC_90_ (MIC/MEC causing inhibition of 90% of the isolates tested) were determined. For calculation purposes, the MIC or MEC values that exceeded the maximum concentration tested were transformed to the next dilution (i.e., if MIC/MEC was >16 mg/L, it was expressed as 32 mg/L) and values that were less than or equal to the minimum concentration tested were transformed to equal (i.e., if MIC/MEC was ≤0.03 mg/L, it was expressed as 0.03 mg/L). MIC_50_/MEC_50_ and MIC_90_/MEC_90_ were only calculated for species from which five or more isolates were tested.

## 3. Results and Discussion

Table 1 shows the GM, MIC_50_/MEC_50_, and MIC_90_/MEC_90_, and the ranges for all the species tested for each testing method at 48 h of incubation. Control strains were within the accepted ranges according to EUCAST and CLSI QC ranges for amphotericin B, voriconazole, and micafungin.

Ibrexafungerp was in vitro active against azole susceptible and resistant *A. fumigatus s.s.* strains. Even though each Cyp51A mutation is linked to a different azole resistance profile [29], ibrexafungerp exhibited encouraging activity against all the Cyp51A mutant strains tested. While MIC_50_ values for voriconazole were 2 mg/L by EUCAST and 0.5 mg/L by CLSI for the susceptible ones and 4 mg/L by both testing methodologies for the resistant strains, MEC_50_s for ibrexafungerp were 0.03 mg/L (EUCAST) and 0.06 mg/L (CLSI). These results are in agreement with those previously reported after in vitro testing [14,15,16,17] and after assessing in vivo activity when administered orally or intravenously in murine models of infection of invasive aspergillosis caused by *A. fumigatus* [20,22].

All cryptic species from the *A. fumigatus* complex tested showed different resistance profiles against amphotericin B and/or voriconazole, something that has been previously documented [3]. However, ibrexafungerp yielded low MECs (GM values were ≤0.227 mg/L) only comparable to those from micafungin (GMs ≤ 0.021 mg/L) against *Aspergillus thermomutatus*, *Aspergillus udagawae*, *Aspergillus hiratsukae*, and *Aspergillus felis*, and even against *Aspergillus lentulus* and *Aspergillus fumigatiaffinis*, which have been described as resistant to more than one of the available antifungal classes [3].

Species belonging to the *Aspergillus terreus* complex are characterized for exhibiting low susceptibility to amphotericin B and sometimes moderate susceptibility to azoles [30]. This statement is in line with the MIC values for the strains here tested, which were the highest of those antifungals tested, followed by voriconazole. GM values for ibrexafungerp ranged from 0.030 mg/L to 0.078 mg/L against the four species tested by EUCAST and CLSI. Micafungin was also active against them, showing GMs lower than 0.044 mg/L. These values are in agreement with those from previous reports on the in vitro activity of this echinocandin against *A. terreus* complex species [31].

The new drug displayed an intermediate in vitro activity against most of the strains from the *Aspergillus ustus* complex tested, whose species are known for yielding intrinsically high MICs to most classes of antifungal drugs [32]. While voriconazole was ineffective (MICs ≥ 8 mg/L) and micafungin revealed good activity (GM values lower than 0.120 mg/L) against them, ibrexafungerp exhibited MEC values (range 0.12–4 mg/L) similar to MICs for amphotericin B (range 0.12–2 mg/L). However, the three *Aspergillus insuetus* strains tested showed higher MECs for this new compound, especially by EUCAST methodology (GM = 3.175 mg/L versus 1.260 mg/L by CLSI). Micafungin (GM values of 0.120 mg/L and 0.095 mg/L by EUCAST and CLSI, respectively) and even amphotericin B (GM = 1.000 mg/L by EUCAST and 0.500 mg/L by CLSI) revealed a better in vitro activity against these isolates than ibrexafungerp. A higher number of strains from this species should be further evaluated.

*Aspergillus ochraceus* and *Aspergillus sclerotiorum*, which are the most commonly isolated species from section Circumdati from immunosuppressed patients, showed a reduced susceptibility for amphotericin B (MIC_50_ of 32 mg/L) and were only moderately inhibited by voriconazole (MIC_90_ of 1 mg/L and 2 mg/L for A. *ochraceus* by EUCAST and CLSI, respectively, and 4 mg/L for *A. sclerotiorum* using both mothodologies), as previously reported [33]. While ibrexafungerp was active against them, with MEC_50_ values of 0.03–0.12 mg/L, micafungin appeared to be the most effective antifungal in vitro of those tested (MEC_50_ values ranging from 0.004 mg/L to 0.015 mg/L).

In agreement with previous studies [3,34], *Aspergillus alliaceus* (*flavus* complex) and *Aspergillus tubingensis* (*niger* complex) show a variable susceptibility against azoles; *A. alliaceus* also yielding elevated MICs against amphotericin B. Ibrexafungerp was mostly inactive against *A. alliaceus*, as 75% (by EUCAST) and 40% (by CLSI) of the total number of isolates tested showed MECs > 2 mg/L. When tested in vitro against *A. flavus* strains in previous studies, this new drug did not display a homogenous effectiveness, being reported as active in some studies, with MEC values ranging from 0.06 mg/L to 0.25 mg/L [14,16], but as inactive in another (MEC range 2–16 mg/L) [15]. Nevertheless, ibrexafungerp exhibited activity against *A. tubingensis* (MEC_50_ was 0.006 mg/L by both methods), although micafungin was the antifungal showing the lowest MECs (MEC_50_s = 0.007 mg/L).

Even though ibrexafungerp was generally active against *Aspergillus* cryptic species and azole susceptible and resistant *A. fumigatus s.s.* strains, micafungin showed the lowest MEC values against most of the species tested in this study. These results are supported by those obtained in previous works involving in vitro testing of ibrexafungerp and caspofungin against *Aspergillus* spp. [15,16]. However, ibrexafungerp has a longer half-life and is available as an oral formulation due to its improved pharmacokinetics and pharmacodynamics [35]. In addition to its good in vitro activity against susceptible and azole resistant *Aspergillus* spp., ibrexafungerp has proven to be effective against a caspofungin resistant *A. fumigatus* isolate [15] and has also yielded good results in neutropenic mice with invasive aspergillosis caused by azole susceptible and resistant *A. fumigatus* [20,22]. Therefore, this drug is currently undergoing phase II as an oral formulation for the treatment of invasive aspergillosis, as well as being in phase III for the treatment of vulvovaginal and invasive candidiasis. Ibrexafungerp has also been tested in combination with other antifungals against a wide range of fungal pathogens [14], showing synergistic interactions both in vitro and in rabbit models when combined with azoles, especially with isavuconazole, against *Aspergillus* spp. [22,36].

In this study, CLSI yielded, in general, lower MICs/MECs than EUCAST. These differences were in most cases higher than 2 dilutions for amphotericin B and ibrexafungerp, while voriconazole and micanfungin showed more comparable results. The differences in the size of the inoculum and in the composition of the media in both methodologies could explain these discrepancies [37]. Besides, MECs were difficult to determine for some strains, which led to discrepancies between CLSI and EUCAST methods. This could cause interpretation problems on a routine daily basis and should be further analyzed by performing multicenter studies on the antifungal susceptibility profile of these species.

Given the encouraging in vitro results displayed by ibrexafungerp against *Aspergillus* spp., and the advantages that this new drug offers in terms of dose administration and improved PK/PD characteristics, further research is warranted in order to complete the clinical trial phases for the treatment of invasive aspergillosis, as well as a multicenter study to establish breakpoints and epidemiological cut off values (ECOFFs) for the main clinically important species of this genus.

## Figures and Tables

**Table 1:** MIC values and ranges for amphotericin B and voriconazole, and MEC values for micafungin and ibrexafungerp against azole–susceptible and resistant *A. fumigatus* isolates and cryptic species of *Aspergillus*, as determined by the CLSI and EUCAST broth microdilution methods.

		Test Method							
		Antifungal Test at 48 h of Incubation by EUCAST and by CLSI							
		EUCAST				CLSI			
Species (no. tested)		AMB	VRC	MCF	IBF	AMB	VRC	MCF	IBF
***Aspergillus fumigatus* complex**									
*Aspergillus fumigatus s.s.* WT (10)	GM	0.435	1.231	0.011	0.040	0.232	0.660	0.005	0.040
	MIC_50_/MEC_50_	0.5	2	0.015	0.03	0.25	0.5	0.004	0.03
	MIC_90_/MEC_90_	0.5	2	0.03	0.12	0.25	2	0.015	0.12
	Range	0.25–0.5	0.5–2	0.004–0.03	0.03–0.12	0.12–0.25	0.25–2	0.004–0.015	0.03–0.12
*Aspergillus fumigatus s.s.* azole R (10)	GM	0.406	2.144	0.013	0.092	0.161	2.000	0.004	0.056
	MIC_50_/MEC_50_	0.5	4	0.015	0.06	0.12	4	0.004	0.06
	MIC_90_/MEC_90_	1	16	0.03	2	0.25	16	0.004	0.12
	Range	0.25–1	0.25–16	0.007–0.06	0.03–8	0.12–0.25	0.25–16	0.004–0.007	0.03–0.12
*Aspergillus lentulus* (20)	GM	3.864	2.639	0.009	0.086	0.636	2.378	0.004	0.074
	MIC_50_/MEC_50_	2	4	0.007	0.06	0.5	2	0.004	0.06
	MIC_90_/MEC_90_	32	4	0.015	0.25	1	4	0.004	0.25
	Range	1–32	0.5–16	0.004–0.03	0.03–0.5	0.12–2	1–4	0.004–0.004	0.03–0.25
*Aspergillus fumigatiaffinis* (10)	GM	22.627	4.287	0.015	0.227	2.144	2.639	0.004	0.040
	MIC_50_/MEC_50_	32	4	0.015	0.12	2	2	0.004	0.03
	MIC_90_/MEC_90_	32	8	0.03	16	4	4	0.004	0.06
	Range	8–32	2–8	0.007–0.03	0.03–16	1–8	2–4	0.004–0.004	0.03–0.06
*Aspergillus thermomutatus* (10)	GM	0.536	2.297	0.021	0.130	0.131	1.741	0.013	0.056
	MIC_50_/MEC_50_	0.5	2	0.015	0.12	0.12	2	0.015	0.06
	MIC_90_/MEC_90_	2	4	0.06	0.5	0.5	4	0.06	0.12
	Range	0.25–2	0.5–4	0.015–0.06	0.03–2	0.03–1	0.25–4	0.004–0.06	0.03–0.12
*Aspergillus udagawae* (10)	GM	3.031	2.000	0.008	0.183	0.616	1.866	0.004	0.069
	MIC_50_/MEC_50_	2	2.000	0.007	0.12	0.5	2	0.004	0.12
	MIC_90_/MEC_90_	8	4.000	0.015	8	1	2	0.004	0.25
	Range	2–16	1–4	0.004–0.015	0.03–8	0.5–1	1–2	0.004–0.007	0.03–0.25
*Aspergillus hiratsukae* (7)	GM	1.641	1.811	0.008	0.221	0.301	1.641	0.005	0.042
	MIC_50_/MEC_50_	1	1	0.007	0.12	0.25	2	0.004	0.06
	MIC_90_/MEC_90_	16	8	0.03	8	2	8	0.015	0.06
	Range	0.5–32	0.5–8	0.004–0.03	0.03–16	0.03–2	0.5–8	0.004–0.015	0.03–0.06
*Aspergillus felis* (2)	GM	1.414	8.000	0.007	0.085	0.707	8.000	0.004	0.060
	Range	1–2	8–8	0.007–0.007	0.06–0.12	0.5–1	8–8	0.004–0.004	0.06–0.06
***Aspergillus terreus* complex**									
*Aspergillus citrinoterreus* (8)	GM	3.668	0.595	0.012	0.078	1.000	0.354	0.010	0.050
	MIC_50_/MEC_50_	4	0.5	0.015	0.06	1	0.5	0.007	0.06
	MIC_90_/MEC_90_	8	1	0.015	0.12	1	0.5	0.03	0.06
	Range	2–16	0.5–1	0.007–0.015	0.06–0.12	1–1	0.25–0.5	0.007–0.03	0.03–0.06
*Aspergillus carneus* (4)	GM	1.189	1.000	0.044	0.030	0.420	1.682	0.004	0.030
	Range	1–2	1–1	0.004–4	0.03–0.03	0.25–0.5	1–2	0.004–0.004	0.03–0.03
*Aspergillus aureoterreus* (3)	GM	2.520	0.794	0.005	0.030	0.621	0.315	0.004	0.048
	Range	0.5–8	0.5–1	0.004–0.007	0.03–0.03	0.12–2	0.25–0.5	0.004–0.004	0.03–0.06
*Aspergillus hortai* (3)	GM	2.000	1.000	0.012	0.030	0.397	0.630	0.004	0.030
	Range	1–4	1–1	0.007–0.015	0.03–0.03	0.25–0.5	0.5–1	0.004–0.004	0.03–0.03
***Aspergillus ustus* complex**									
*Aspergillus calidoustus* (15)	GM	0.955	8.378	0.115	0.952	0.395	8.378	0.072	0.500
	MIC_50_/MEC_50_	1	8	0.12	1	0.5	8	0.06	0.5
	MIC_90_/MEC_90_	2	16	0.25	4	1	8	0.12	1
	Range	0.25–2	4–16	0.06–0.25	0.12–4	0.12–1	8–16	0.03–0.12	0.25–1
*Aspergillus insuetus* (3)	GM	1.000	8.000	0.120	3.175	0.500	12.699	0.095	1.260
	Range	1–1	8–8	0.12–0.12	2–8	0.5–0.5	8– 1 6	0.06–0.12	0.5–2
*Aspergillus keveii* (2)	GM	0.707	16.000	0.085	0.707	0.354	16.000	0.030	1.000
	Range	0.5–1	16–16	0.06–0.12	0.5–1	0.25–0.5	16–16	0.03–0.03	0.5–2
***Aspergillus* section Circumdati**									
*Aspergillus ochraceus* (10)	GM	10.556	0.871	0.017	0.121	1.414	0.707	0.013	0.122
	MIC_50_/MEC_50_	32	1	0.015	0.12	2	1	0.015	0.12
	MIC_90_/MEC_90_	32	1	0.06	0.5	2	1	0.03	0.25
	Range	2–32	0.5–1	0.007–0.06	0.03–1	1–2	0.25–1	0.004–0.03	0.03–0.25
*Aspergillus sclerotiorum* (5)	GM	16.000	2.297	0.007	0.034	2.297	1.741	0.006	0.106
	MIC_50_/MEC_50_	32	2	0.007	0.03	2	2	0.004	0.12
	MIC_90_/MEC_90_	32	4	0.015	0.06	4	4	0.015	0.5
	Range	4–32	1–4	0.004–0.015	0.03–0.06	2–4	1–4	0.004–0.015	0.03–0.5
***Aspergillus flavus* complex**									
*Aspergillus alliaceus* (20)	GM	28.840	0.420	0.029	5.077	17.148	0.248	0.007	1.506
	MIC_50_/MEC_50_	32	0.5	0.03	16	32	0.250	0.007	1
	MIC_90_/MEC_90_	32	1	0.06	32	32	1	0.015	16
	Range	4–32	0.12–1	0.015–0.06	0.03–32	1–32	0.06–1	0.004–0.03	0.12–16
***Aspergillus niger* complex**									
*Aspergillus tubingensis* (16)	GM	0.249	1.044	0.010	0.065	0.089	1.682	0.007	0.053
	MIC_50_/MEC_50_	0.25	1	0.007	0.06	0.12	2	0.007	0.06
	MIC_90_/MEC_90_	0.25	2	0.03	0.12	0.12	2	0.015	0.06
	Range	0.12–0.5	0.5–2	0.007–0.03	0.03–0.12	0.06–0.12	1–2	0.004–0.03	0.03–0.25
**All (168)**	GM	2.246	1.736	0.019	0.329	0.640	1.465	0.017	0.185
	MIC_50_/MEC_50_	2	2	0.015	0.12	0.5	2	0.015	0.12
	MIC_90_/MEC_90_	32	8	0.12	8	4	8	0.06	1
	Range	0.06–32	0.12–16	0.007–4	0.06–32	0.03–32	0.06–16	0.004–0.12	0.03–16

AMB, amphotericin B; VRC, voriconazole; MCF, micafungin; IBF, ibrexafungerp; *s.s.*, *sensu stricto*.

## Data Availability

Not applicable.
